# A comprehensive integrated disease management program for phenylketonuria (IDMP-PKU) from Türkiye: rationale, design and patient characteristics

**DOI:** 10.1186/s13023-025-03702-7

**Published:** 2025-08-01

**Authors:** Mehmet Cihan Balci, Deniz Kor, Yilmaz Yildiz, Meryem Karaca, Fatma Derya Bulut, Ayca Burcu Kahraman, Alihan Yesil, Ezgi Burgac, Kismet Ciki, Arzu Selamioglu, Burcu Koseci, Asli Durmus, Irem Kaplan, Esra Kara, Halise Neslihan Mungan, Serap Sivri, Gulden Fatma Gokcay, Aysegul Tokatli, Mubeccel Demirkol, Turgay Coskun, Imran Ozalp

**Affiliations:** 1 Division of Pediatric Nutrition and Metabolism, Istanbul Medical Faculty, Children’s Hospital, Istanbul University, Turgut Ozal Millet Cd, Fatih, 34093 Istanbul, Türkiye; 2 Division of Pediatric Metabolism and Nutrition, Department of Pediatrics, Faculty of Medicine, Cukurova University, Adana, Türkiye; 3 Division of Pediatric Metabolism, Department of Pediatrics, Faculty of Medicine, Hacettepe University, Ankara, Türkiye

**Keywords:** Phenylketonuria, Newborn screening, Diagnosis, Allelic variant, Consanguinity, Maternal education

## Abstract

**Background:**

Phenylketonuria is an autosomal recessive disorder characterized by the deficiency of phenylalanine hydroxylase, which converts phenylalanine into tyrosine. Diagnosis and prompt initiation of appropriate treatment shortly after birth are important for achieving optimal outcomes in phenylketonuria. IDMP-PKU is an ongoing study to gain insight into the patient journey and identify the unmet needs and areas for improvement in diagnosis, treatment, and follow-up of PKU in Türkiye.

**Aim:**

To present the rationale and design of the IDMP-PKU study, as well as the findings from an interim analysis, describing baseline demographic, diagnosis, family history, and genetic testing data for 1553 children enrolled in the study.

**Method:**

This is a multicenter, observational registry-based study, conducted in 3 tertiary pediatric metabolic clinics in Türkiye. The study provides a descriptive analysis of baseline demographic, diagnosis, family history, and genetic testing data of study population.

**Results:**

The study included 1,553 patients (median age: 10 (IQR 5–18) years; 37.1% classical PKU) from 90% of the cities in Türkiye, diagnosed between 1981 and 2022. Parental consanguinity was reported in 43.5% of families (27.1% first cousins). The most frequently detected allelic variant was c.1066-11G > A (IVS-10-11G > A) (22.8%). Homozygous mutations were more common in patients with parental consanguinity (76.8% vs 17.1%; *p* < 0.001). The median time to diagnosis improved to 21 days after the implementation of the national newborn screening (NBS) program in December 2006 but 28.6% of patients were diagnosed after one month of age. Low level of maternal education was associated with longer time to diagnosis (*p* < 0.001).

**Conclusions:**

Implementation of national NBS has contributed to earlier identification of patients with PKU. Increasing the number of screening laboratories and pediatric metabolic clinics will speed up the diagnostic process and help achieve the guideline-recommended time for diagnosis and initiation of treatment. In countries with high rates of consanguineous marriages, increasing public awareness of PKU and genetic counselling before marriage will be valuable in reducing the prevalence of PKU.

**Supplementary Information:**

The online version contains supplementary material available at 10.1186/s13023-025-03702-7.

## Introduction

Phenylketonuria (PKU; OMIM # 261600) is an autosomal recessive disorder characterized by the deficiency of phenylalanine hydroxylase (PAH; EC 1.14.16.1), which converts phenylalanine (Phe) into tyrosine (Tyr) [1]. The principal laboratory finding of the disorder is hyperphenylalaninemia (HPA) with an elevated Phe:Tyr ratio [1]. PKU is caused by biallelic pathogenic variants in the *PAH* gene (12q22-q24.2) [1, 2]. As of March 29, 2024, a total of 3,370 *PAH* variants have been identified and registered in the International Phenylalanine Hydroxylase Gene Locus-Specific Database (PAHvdb) [3].

Individuals with PKU appear healthy at birth but, if left untreated, they develop progressive neuropsychiatric signs and symptoms from infancy onwards [4], including microcephaly, seizures, developmental delay, intellectual disability, behavioral, and mood disorders [1, 4, 5]. Early identification of affected individuals and initiation of treatment soon after birth are crucial to minimize the occurrence of adverse outcomes [6–8].

The introduction of the Guthrie test in the early 1960 s, the first newborn screening (NBS) tool to detect HPA, was a major milestone in the diagnosis of PKU [9]. Over time, more sensitive techniques such as high-performance liquid chromatography (HPLC) [10] and tandem mass spectrometry (MS/MS) [11] were developed. Currently, MS/MS is the preferred NBS method worldwide [12], as it is a highly sensitive and specific method that allows screening for multiple metabolic disorders in a single analysis [11, 12].

The severity of the PAH deficiency determines the PKU phenotype which ranges from mild HPA (120–360 µmol/L) to classical PKU (> 1,200 µmol/L) based on the untreated blood Phe levels [8]. A life-long Phe restricted diet is the cornerstone of PKU treatment and recommended in combination with Phe-free amino acid supplements for all patients except those with mild HPA, to keep blood Phe levels within the target ranges [6, 7]. Glycomacropeptide (GMP) [13] or large neutral amino acid (LNAA) supplementation [14] may also be used along with dietary treatment. Sapropterin dihydrochloride, a synthetic BH_4_ for use in all BH_4_ responsive patients [15] and, pegvaliase, a pegylated phenylalanine ammonia lyase (PAL) enzyme that substitutes the deficient PAH activity in adults [16], are the other therapeutic options. Compliance with dietary recommendations, medical nutrition therapy (MNT) and medication, if any, determines blood Phe levels and prognosis. Therefore, individuals with HPA/PKU require a lifelong monitoring of metabolic, nutritional, and psychoneurological status [6, 7].

Türkiye is one of the countries with the highest prevalence of PKU [1, 2, 5] which is around 1 in 5,500 live births [17, 18]. The Association for Screening and Protecting Children with Phenylketonuria launched the PKU Integrated Disease Management Program (IDMP-PKU) in 2020, recognizing the critical role of effective disease management in improving clinical outcomes. As part of this program, an observational study was initiated to gain insight into the patient journey, to identify the unmet needs and areas for improvement in diagnosis, treatment, and follow-up of PKU in Türkiye. The comprehensive scientific data from this study is expected to inform clinical practice and guidelines at the national and global level, contributing to the development of policies that will improve outcomes in HPA/PKU using an evidence-based approach.

In this study, we aim to present the rationale and design of the IDMP-PKU study, as well as the findings from an interim analysis, describing baseline demographic, diagnosis, family history, and genetic testing data for 1553 children enrolled in the study.

## Material and methods

The IDMP-PKU study is collecting data on demographic, clinical, genetic characteristics of patients with HPA/PKU, their management and outcomes in Türkiye. The study aimed to identify the subgroups of HPA due to deficiency of PAH, assess the neurocognitive course of the disease, determine the correlation between genotypes and clinical phenotypes, reveal the similarities and differences between national and global data, identify the key success factors for better prognosis by comparing the outcomes achieved with different treatment modalities, and establish national diagnosis, follow-up, and treatment parameters.

### Study design and settings

The IDMP-PKU is an ambidirectional, multicenter, registry-based observational study conducted in three Pediatric Nutrition and Metabolism Clinics at Istanbul, Hacettepe and Cukurova Universities located in three cities (Istanbul, Ankara and Adana, respectively). The study sites are among the metabolic clinics with the highest HPA/PKU patient populations in the country, treating patients referred from various geographical regions of the country.

All participants in the study received treatment and monitored as per local clinical practice. The planned study period was at least 3 years after the initial ethics committee approval. The first patient was enrolled in May 2021 and data collection was ongoing at the time of the interim analysis data presented here. The timeline showing the key steps in development and conduct of IDMP-PKU is provided in Supplementary Fig. 1.

### Patients

Inclusion criteria were a diagnosis of HPA/PKU due to PAH deficiency, assessment and/or treatment for HPA/PKU at least once at the study center and written informed consent for data collection provided by the patient and/or the parent or guardian. Patients with transient HPA or BH_4_ metabolism disorders or other clinical conditions associated with intellectual disability, and those participating in other studies were excluded.

### Assessments

Data on key demographics (age, sex, educational status, working status, marital status, birthplace), family history (number of children in family, number of siblings with HPA/PKU, parental consanguinity, parental history of HPA/PKU, educational status of parents), diagnostic methods and clinical characteristics (age at diagnosis of HPA/PKU, mode of diagnosis, blood Phe and Tyr levels, *PAH* genotypes were collected.

Patients were classified into four phenotypes based on their blood Phe levels in the untreated state in accordance with the criteria outlined in a recent paper on recommendations for the treatment of PKU from Türkiye: mild HPA (120–360 µmol/L), mild PKU (360–600 µmol/L), moderate PKU (600–1,200 µmol/L), and classical PKU (> 1,200 µmol/L) [8].

### Statistics

All statistical analyses were conducted using SPSS version 20 software. Demographic and clinical characteristics were analyzed using descriptive statistics. Continuous variables were summarized as mean, standard deviation (SD), minimum – maximum or median and interquartile range (IQR 25%–75%) values. Categorical variables were summarized as frequencies and percentages. The normality of the data was checked using graphical and analytical methods. Non-normally distributed and ordinal data were compared using the Mann–Whitney U test and the Kruskal–Wallis test. Where differences were significant, pairwise comparisons were made using the Mann–Whitney U test and interpreted using the Bonferroni correction. In correlational analyses, correlation coefficients and statistical significance were calculated using the Pearson test for normally distributed data and the Spearman test for ordinal or non-normally distributed data. A *p*-value of < 0.05 was considered statistically significant.

## Results

A total of 1,553 patients enrolled in the IDMP-PKU study as of April 30 th, 2023 were included in this interim analysis set.

### Demographics

The median (IQR25%–75%) age of the study population was 10 [5–18] years and 52.1% (*n* = 809) of patients were female. A total of 1,049 (67.5%) participants had graduated from or were attending a regular educational institution and 115 (7.4%) had a history/need of special education due to PKU-related disabilities (Table 1). More than two-thirds (70.4%) of 514 patients of working age (15–64 years), were neither in education nor in employment (Table 1).

**Table 1:** Key patient characteristics

		*n* (%)	Mean ± SD (Min–Max) or Median (IQR 25%–75%)
Sex (*n* = 1,553)	*Female* *Male*	809 (52.1) 744 (47.9)	
Age (years) (*n* = 1,553)			10 (5–18)
	*0–1*	43 (2.8)	
	*1–3*	167 (10.8)	
	*3–5*	256 (16.5)	
	*5–10*	286 (18.4)	
	*10–18*	389 (25)	
	≥ *18*	412 (26.5)	
History of education (*n* = 1,553)	*Regular* *Special*	1049 (67.5)^*^ 115 (7.4)	
Working status (*n* = 514)^**^	*Registered* *Unregistered* *Not working*	81 (15.8) 71 (13.8) 362 (70.4)	
Age at diagnosis of HPA/PKU (days) (*n* = 1,522)			21 (13–40)
Time from birth to HPA/PKU diagnosis (*n* = 1,522)	*First 7 days* *8–15 days* *16*–*30 days* *1*–*3 months* *4*–*6 months* *6 months*–*1 year* *1*–*3 years* *4*–*6 years* *7–10 years* *11–18 years* ≥ *18 years*	121 (8) 370 (24.3) 527 (34.6) 330 (21.7) 44 (2.9) 46 (3) 43 (2.8) 12 (0.8) 10 (0.7) 13 (0.9) 6 (0.4)	
Mode of HPA/PKU diagnosis (*n* = 1,550)	*Newborn screening* *Symptom-based* *Family screening* *Family history*	1,328 (85.7) 104 (6.7) 101 (6.5) 17 (1.1)	
Pre-treatment Phe level (µMol/L) (*n* = 1,534)			805.7 (311.85–1,558.65)
Clinical phenotype^***^ (*n* = 1,534)	*Mild HPA* *(*≥ *120–* < *360 µMol/L)*	452 (29.5)	222.76 ± 68.7 (120–357.6)
	*Mild PKU* *(*≥ *360–* < *600 µMol/L)*	201 (13.1)	454.67 ± 69.51 (360–597.28)
	*Moderate PKU* *(*≥ *600–* < *1,200 µMol/L)*	307 (20)	878.01 ± 183.2 (600–1,198.8)
	*Classical PKU* *(*≥ *1,200 µMol/L)*	574 (37.4)	1866.07 ± 498.91 (1,200–4,351.8)

HPA, Hyperphenylalaninemia; IQR: Interquartile range; n: Number of patients; Phe: Phenylalanine; PKU: Phenylketonuria. ^*^409 of the remaining 504 participants were below compulsory school age for children (5 years) ^**^Working age (15–64 years) population excluding students, ^***^Based on pre-treatment blood Phe levels

### Family history

There was a total of 1,398 families whose children participated in the study. 20.7% of the families had more than 1 child with HPA/PKU (mean: 1.22 ± 0.47; min–max: 1–5) (Table 2 and Supplementary Table 1). Of the 922 mothers and 791 fathers who had been evaluated for blood Phe levels, 12% (*n* = 111) and 9.4% (*n* = 74) had HPA, respectively (Table 2).

**Table 2:** Patients’ family histories

		*n* (%)	Mean ± SD (Min–Max)
HPA in mother (*n* = 922)	Yes No	111 (12) 811 (88)	
HPA in father (*n* = 791)	Yes No	74 (9.4) 717 (90.6)	
Parental consanguinity (*n* = 1,380)	Yes	600 (43.5)	
	First cousins	374 (27.1)	
	Second cousins	79 (5.7)	
	Distant relatives	147 (10.7)	
	No	780 (56.5)	
Number of children with HPA in the family (*n* = 1,398)			1.22 ± 0.47 (1–5)
	1	1,109 (79.3)	
	2	266 (19)	
	3	21 (1.5)	
	4	1 (0.1)	
	5	1 (0.1)	

HPA, Hyperphenylalaninemia; IQR, Interquartile range

Parental consanguinity was reported in 43.5% of 1,380 families, with 27.1% of parents being first cousins (Table 2). The parents had consanguinity in 40%, 55% and 76% of families with one, two and three children with HPA/PKU, respectively. Two families, one with four and the other with five children affected by HPA/PKU, had no history of parental consanguinity (Supplementary Table 2).

### PKU phenotype

Classical PKU (37.4%) was the most common phenotype in the study population followed by mild HPA (29.5%). The median Phe level at baseline was 805.7 (311.85–1,558.65) µMol/L (Table 1). There was a relationship between parental consanguinity and the PKU phenotype. History of parental consanguinity was less common in patients with mild HPA (30.8%) and mild PKU (30.9%) compared to those with moderate PKU (52.2%) and classical PKU (52.9%) (*p* < 0.001) (Table 3).

**Table 3:** Association between phenotypes and parental consanguinity

		Parental consanguinity *n*(%)		Total	*p* value
		Yes	No		
Phenotype classified based on untreated Phe level	Mild HPA	122 (30.8)	274 (69.2)	396	< 0.001^a^
	Mild PKU	54 (30.9)	121 (69.1)	175	
	Moderate PKU	143 (52.2)	131 (47.8)	274	
	Classical PKU	276 (52.9)	246 (47.1)	522	
Total		595 (43.5)	772 (56.5)	1,367 (100)	

HPA, Hyperphenylalaninemia; PKU, Phenylketonuria. ^a^Chi-square test

### Diagnostic methods

**Fig. 1:**
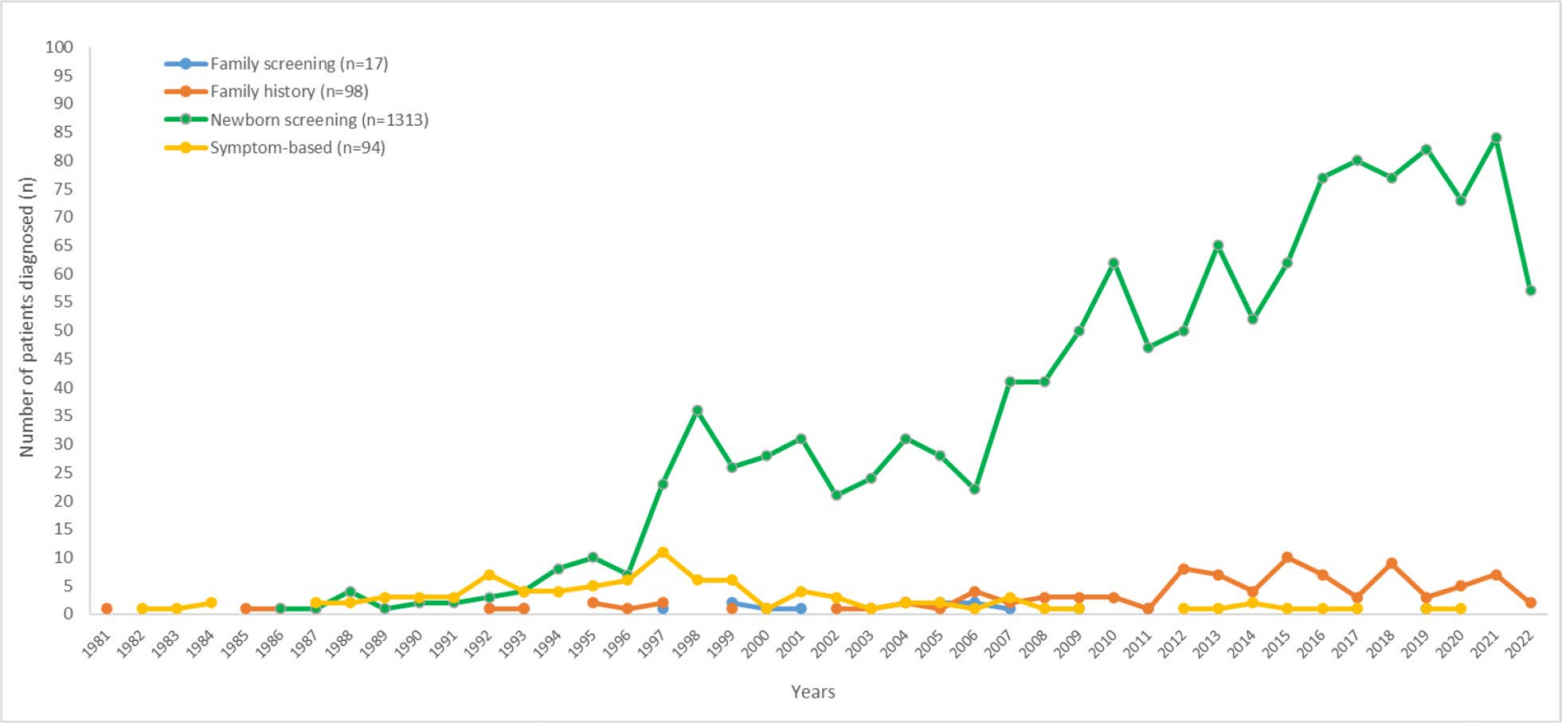
Method of HPA (Hyperphenylalaninemia)/PKU (Phenylketonuria) diagnosis (*n* = 1,522). Number of newly diagnosed patients has increased as of the initiation of pilot newborn screening program (NBS) in 1985. A prominent increase occurred after the implementation of national NBS in December 2006 (colour should be used for this figure)

Overall, 85.7% of patients (*n* = 1,328) were diagnosed based on an abnormal NBS result for PKU in the study (Table 1). Among the 1,522 patients whose dates of diagnosis are known, 422 (27.7%) were diagnosed during the pilot NBS program for PKU, which ran from 1985 to 2006 and 1,095 (71.9%) were diagnosed following the initiation of the national NBS in December 2006 in Türkiye. There were five patients in the study population who were diagnosed before the year 1985: four based on symptoms and one based on family history. After the launch of pilot NBS, NBS became the primary tool for identifying patients with HPA/PKU and the number of new diagnoses markedly increased as of 2007 (Fig. 1).

The three most common methods for initial diagnosis of PKU after the implementation of pilot NBS program were HPLC- serum/plasma (52%; 539/1,037 patients), HPLC-dried blood spot (24.6%; 255/1,037 patients) and MS/MS (11.8%; 122/1,037 patients). Prior to the availability of these methods for NBS in Türkiye, semi-quantitative tests, namely the Guthrie test and the Thin Layer Chromatography (TLC), were the primary diagnostic tools employed in the initial diagnosis of PKU.

### Time to diagnosis

**Fig. 2:**
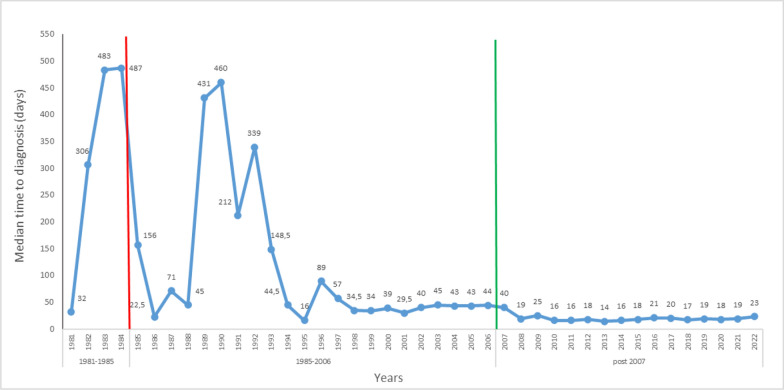
Time to HPA/PKU diagnosis by year of diagnosis (days) (*n* = 1522). Red and green lines in the figure denote the introduction of the pilot and national NBS for PKU. (colour should be used for this figure)

The median age at diagnosis was as high as 483 days before the implementation of NBS program. The median time to HPA/PKU diagnosis exhibited a progressive decline following the expansion of the pilot NBS program but remained at approximately 20 days even after the establishment of the national NBS. (Fig. 2).

Patients who underwent NBS were most frequently diagnosed (38.8%) 16 to 30 days after birth (median: 21 days; IQR 25–75:18–25 days) While 5.6% of patients were diagnosed within the first week of their birth, 28.6% of patients were diagnosed beyond one month (Supplementary Table 3).

The median time to PKU diagnosis differed significantly between the study sites and was shorter for Istanbul University compared to the other sites (18 days for Istanbul University vs 23 days for both Hacettepe and Cukurova Universities; *p* < 0.001) (Table 4).

**Table 4:** Time to HPA/PKU diagnosis by study site (*n* = 1,522)

Study site	*n* (%)	Time to diagnosis (day) Median (IQR 25%−75%)	*p* value
Istanbul University, Istanbul Faculty of Medicine	515 (33.8)	18 (12–36)	< 0.001^a^
Hacettepe University, Faculty of Medicine	616 (40.5)	23 (13–44)	
Cukurova University, Faculty of Medicine	391 (25.7)	23 (15–37)	

HPA, Hyperphenylalaninemia; IQR, Interquartile range; n: Number of patients; PKU, Phenylketonuria. ^a^Kruskal-Wallis test

The median time to diagnosis with newborn screening varied between PKU subtypes from 18–24 days. (*p* = 0.002) (Table 5). While the clinical phenotypes were comparable in terms of time to diagnosis during the pilot NBS, a significant difference was observed after the introduction of the national program, with PKU patients being diagnosed earlier (16 days for classical PKU and 17 days for mild and moderate PKU) compared to patients with HPA (23 days) (*p* < 0.001) (Supplementary Table 4).

**Table 5:** Phenotypes and time to diagnosis in patients who underwent NBS (*n* = 1,302)^a^

Phenotype	Phe level (µMol*/L)*	*n* (%)	Time to diagnosis (day) Median (IQR 25%−75%)	*p* value
HPA	(120 ≤ x < 360)	413 (31.7)	24 (16–32)	0.002^b^
Mild PKU	(360 ≤ x < 600)	165 (12.7)	19 (12–27.5)	
Moderate PKU	(600 ≤ x < 1,200)	233 (17.9)	20 (12–42.5)	
Classical PKU	x ≥ 1,200	491 (37.7)	18 (13–34)	

^a^Based on untreated Phe levels, ^b^Kruskal-Wallis test HPA, Hyperphenylalaninemia; IQR, interquartile range; n: number of patients; PKU, Phenylketonuria

The time to diagnosis was significantly longer in participants with a low maternal education level. (Supplementary Table 5) The paternal educational level did not appear to influence the time to diagnosis.

### PAH variants

In total, 1,976 PKU alleles were identified in 1,017 patients with genotype data. These included 137 distinct allelic variants: 117 substitutions (85.5%), 17 deletions (12.3%), 2 duplications (1.4%) and 1 insertion (0.7%). Homozygous mutations were detected in 429 (42.2%) patients. Homozygous mutations were more common in patients with parental consanguinity than in those without (76.8% vs 17.1%; *p* < 0.001). While homozygous mutations were detected in 84% and 69.6% of patients born from first and second cousin parents, respectively, compound heterozygous mutations predominated in patients with unrelated parents (Supplementary Table 6).

c.1066-11G > A (IVS-10-11G > A) was the most commonly detected variant (22.8%) in the study followed by c.782G > A (p.Arg261Gln) (9.2%) and c.898G > T (p.Ala300Ser) (6.2%).The most frequently observed (> 5%) allelic variants in the study population accounted for 49.1% of the identified *PAH* mutations (Supplementary Table 7).

## Discussion

This article presents an interim analysis of the baseline demographic, diagnosis, family history and genetic testing data of a cohort of 1,553 individuals with HPA/PKU in Türkiye, which is among the countries with the highest PKU prevalence globally. The study revealed that the national NBS program the facilitated the earlier identification of patients with HPA/PKU. Nevertheless, the age at which the disease was diagnosed remained above the recommended threshold for initiating treatment in most cases. The high prevalence of homozygous PAH variants and severe disease phenotypes due to high rates of parental consanguinity, and the association between a low level of maternal education and a delay in diagnosis were the other noteworthy findings of the study.

In Türkiye, NBS for PKU began in the 1980 s as a pilot project at Hacettepe University, one of the sites of the IDMP-PKU study. It has been carried out as part of the national NBS program since December 2006 [19]. All heel-prick test samples collected across the country are centrally analyzed at two laboratories in Istanbul and Ankara, designated by the Ministry of Health. Infants with a presumptive positive screening test result are referred for further evaluation to pediatric metabolic clinics that are currently available in 22 cities [20]. According to the most recent data, the NBS program screened 97.2% of all live births in 2021, and 196 babies were diagnosed with PKU [18].

The IDMP-PKU study is being conducted in three pediatric metabolic clinics located in the first, second and seventh largest cities in Türkiye, representing approximately 28% of the country’s population [21]. The study sites are among the reference metabolic clinics for patients with HPA/PKU and serve patients from almost all geographical locations across the country. In the present study, the time to diagnosis of HPA/PKU exceeded the first 7 to 10 days of life in a significant number of patients, which is the period recommended by the American College of Genetics and Genomics (ACMG) [6], European guidelines [7], and Turkish medical experts [8] for the initiation of treatment. In the IDMP-PKU study, HPA/PKU was diagnosed within the first week of life in only 8% of patients, while two-thirds of patients were diagnosed after one month of age. This finding is of particular importance, given that previous research indicated that each four-week delay in the initiation of PKU treatment was associated with a decline of four points in the intelligence quotient (IQ) [22].

Late diagnosis of HPA/PKU is a pervasive issue, reported even in countries with established NBS programs [23–27]. In an international survey of more than 8,500 patients with PKU and 60 health care providers (HCPs) from 31 clinics in 15 Southern and Eastern Europe countries, 41.9% of centers reported reaching children with positive screening tests for PKU within the first 9 days after birth, while 25% reported reaching them between 10 and 15 days after birth [23]. Early diagnosis rates were higher in the NBS-PKU Connect web-based patient registry from the US where 49.3% of survey respondents were diagnosed within the first week after birth and 36% during the second week of life [24]. Consistent with our findings, several recent observational studies from Türkiye pointed out to a delay in diagnosis and treatment initiation in patients with PKU [28, 29]. The observation that the time to diagnosis has remained constant at around 20 days over the past decade in the IDMP-PKU study necessitates a comprehensive understanding of the underlying causes in order to provide effective solutions to overcome this issue. The presence of central NBS laboratories in only two cities (Ankara and Istanbul) and the requirement by the Ministry of Health for repeat sampling and analysis for patients with initial Phe levels of 2–4 mg/dL are the main factors delaying a definite diagnosis of HPA/PKU in Türkiye. Furthermore, the insufficiency in the number and distribution of pediatric metabolic clinics across the country causes delays in evaluation of patients with presumptive screening test results [20]. Very recently, a study by Kadıoğlu Yılmaz & Bağcı [29] from Türkiye reported that refugee status, the need for repeated heel-prick tests, an initial Phe level below 240 µmol/L, and being born to consanguineous parents were associated with delays in diagnosis. The combined prevalence of patients with mild PKU and mild HPA > 40% may explain, at least in part, the high rates of late diagnosis in the IDMP-PKU cohort. Furthermore, The IDMP-PKU study showed that the low level of maternal education was associated with delays in diagnosis. Increasing mothers’ awareness and knowledge of the rationale, method, benefits, and potential consequences of NBS, commencing from the antenatal period, is therefore essential to enhance the effectiveness of an NBS program as previously highlighted in several studies [29–32]. A recent multicenter study in Eastern and Northern Türkiye found that knowledge and attitudes about NBS were inadequate among mothers in nuclear families, those with a single child, and those who had received regular antenatal care [33]. We believe that informing mothers about NBS is of paramount importance in rural areas, where maternal educational levels are low, home births are common, and the risk of inherited diseases is high due to the predominance of consanguineous marriages.

A previous research in Türkiye reported an association between refugee status and delayed diagnosis of PKU [29]. The IDMP-PKU study does address this issue However, given that Türkiye has been exposed to a significant immigration over the past decade, it might have an impact on the findings of the study. In contrast to global data [34], several studies conducted in Türkiye have consistently reported that the COVID-19 pandemic has not negatively affected NBS in the country [35–37]. Despite the significant disruption to health services during the pandemic, there was no discernible deterioration in the time taken to diagnose PKU in the present study.

We observed a significant difference in age at diagnosis, between the study sites which may be related to geographical, socio-cultural and economic factors. According to the 2018 Türkiye Demographic and Health Survey, the prevalence of consanguineous marriages in Türkiye was 23.5%, with rates exceeding 30% in the Northeastern, Eastern and Southeastern regions of the country [38]. Despite a decline over the recent years, the prevalence of consanguineous marriages remains high, particularly in Eastern and Southeastern regions [39]. Given the established correlation between consanguinity and autosomal recessive disorders [40], it is not surprising that the prevalence of parental consanguinity in the IDMP-PKU study (43.5%) exceeded that observed in the general population. In a single-center study conducted in Southeastern Türkiye, where the rate of consanguineous marriages is highest in the country, Toktas et al. [41] found that 65% of patients with PKU had a history of parental consanguinity. Oz S [42] also reported a high prevalence of parental consanguinity (44.9%) in her observational study of 673 patients with PKU, conducted in a clinic with a large number of patients from Southeastern Anatolia.

“Genotype data were available for two thirds of IDMP-PKU cohort. Despite the heterogeneity of the mutational spectrum in the IDMP-PKU, the five most common allelic variants constituted almost half of the alleles. This may be explained by the high rate of parental consanguinity in the study population. It is noteworthy that, in the present study, homozygous mutations were prevalent among patients born to consanguineous parents. Similar to our observations, a study of 635 patients with PKU in Iran also reported that most patients born to consanguineous parents had homozygous *PAH* mutations [43]. A study from Türkiye has previously reported that 1066-11G > A (IVS-10-11G > A) mutation, the most frequently detected mutation in the present study, was prevalent in patients with classical PKU [44]. Parental consanguinity increases the chance that both parents carry the same recessive gene, thereby raising the likelihood of homozygous mutations in offsprings by inheriting two copies of the same mutation. Consequently, the high prevalence of consanguineous marriages and the presence of mutations associated with the severe form of the disease can explain the high frequency of patients with classical PKU born to consanguineous parents in the present study. Offering genetic counseling to these families deserves special attention to promote healthier generations. The IDMP-PKU study provides a concise overview of the journey of PKU screening in Türkiye, one of the countries with highest PKU prevalence worldwide. The study is distinctive due to the substantial number of participants and the extensive data set, which spans a period of 40 years. Utilizing a dataset comprising over 1500 patient records, the IDMP-PKU study has shown the temporal progression of the diagnostic process following the implementation of the screening program in Türkiye, offering valuable insights into the subsequent steps that need to be taken to achieve further improvement. In this regard, our findings can inform local practices and policies, as well as guide countries planning to implement NBS programs.

### Limitations

The study has several limitations. First, it was conducted in three pediatric metabolic clinics. Investigation of real-life data from a larger number of PKU centers will enrich knowledge of local clinical practice and allow identification of regional differences and unmet needs in the diagnosis and management of PKU across the country. Second, due to the observational nature of the study, the analysis was performed using data available from hospital records and medical histories. The COVID-19 pandemic affected data availability and collection by disrupting clinic visits. Thirdly, the study did not collect information on technical, logistical and resource-related drawbacks of the diagnostic process of the disease, as well as family knowledge of PKU, both of which have an impact on the effectiveness of an NBS program.

## Conclusions

PKU screening, which was introduced in Türkiye in the mid-1980 s and has been part of the national newborn screening program since 2007, has contributed to earlier diagnosis, but has not yet reached the guideline-recommended time for initiation of treatment. Increasing the number of screening laboratories and pediatric metabolic clinics throughout the country will speed up the diagnostic process and ensure earlier treatment initiation. Because of the high rate of consanguineous marriages in Türkiye, public awareness of PKU should be raised, parents should be informed about the process and possible outcomes from pregnancy onwards, and couples should be offered genetic counselling before marriage and pregnancy. The results of this interim analysis of the IDMP-PKU study will add to the scientific data on this rare disease, provide a basis for future studies and be useful in formulating policies and guidelines.

## Supplementary Information

Additional file 1.

Additional file 2.

Additional file 3.

Additional file 4.

Additional file 5.

Additional file 6.

Additional file 7.

Additional file 8.

## Data Availability

The data that support the findings of this study are available from Wellpoint Group companies but restrictions apply to the availability of these data, which were used under license for the current study, and so are not publicly available. Data is, however available from the authors upon reasonable request and with permission of Wellpoint Group companies.

## References

[1] Blau N, van Spronsen FJ, Levy HL. Phenylketonuria. Lancet. 2010;376(9750):1417–27. 20971365 10.1016/S0140-6736(10)60961-0

[2] Hillert A, Anikster Y, Belanger-Quintana A, Burlina A, Burton BK, Carducci C, et al. The genetic landscape and epidemiology of phenylketonuria. Am J Hum Genet. 2020;107(2):234–50. 32668217 10.1016/j.ajhg.2020.06.006 PMC7413859

[3] PAHvdb: Phenylalanine Hydroxylase Gene Locus-Specific Database [Available from: http://www.biopku.org/home/pah.asp.

[4] Stone WL BH, Los E. Phenylketonuria: Treasure Island (FL): StatPearls Publishing; 2024 [StatPearls]. Available from: www.ncbi.nlm.nih.gov/books/NBK535378/.

[5] Elhawary NA, AlJahdali IA, Abumansour IS, Elhawary EN, Gaboon N, Dandini M, et al. Genetic etiology and clinical challenges of phenylketonuria. Hum Genomics. 2022;16(1):22. 35854334 10.1186/s40246-022-00398-9 PMC9295449

[6] Vockley J, Andersson HC, Antshel KM, Braverman NE, Burton BK, Frazier DM, et al. Phenylalanine hydroxylase deficiency: diagnosis and management guideline. Genet Med. 2014;16(2):188–200. 24385074 10.1038/gim.2013.157

[7] van Wegberg AMJ, MacDonald A, Ahring K, Bélanger-Quintana A, Blau N, Bosch AM, et al. The complete European guidelines on phenylketonuria: diagnosis and treatment. Orphanet J Rare Dis. 2017;12(1):162. 29025426 10.1186/s13023-017-0685-2 PMC5639803

[8] Coşkun T, Çoker M, Mungan N, Özel HG, Sivri HS. Recommendations on phenylketonuria in Turkey. Turk J Pediatr. 2022;64(3):413–34. 35899555 10.24953/turkjped.2021.4098

[9] Guthrie R, Susi A. A simple phenylalanine method for detecting phenylketonuria in large populations of newborn infants. Pediatrics. 1963;32:338–43. 14063511

[10] Wu JT. Screening for inborn errors of amino acid metabolism. Ann Clin Lab Sci. 1991;21(2):123–42. 2029175

[11] Chace DH, Millington DS, Terada N, Kahler SG, Roe CR, Hofman LF. Rapid diagnosis of phenylketonuria by quantitative analysis for phenylalanine and tyrosine in neonatal blood spots by tandem mass spectrometry. Clin Chem. 1993;39(1):66–71. 8419060

[12] Millington DS. How mass spectrometry revolutionized newborn screening. J Mass Spectrom Adv Clin Lab. 2024;32:1–10. 38333514 10.1016/j.jmsacl.2024.01.006 PMC10847993

[13] Ney DM, Gleason ST, van Calcar SC, MacLeod EL, Nelson KL, Etzel MR, et al. Nutritional management of PKU with glycomacropeptide from cheese whey. J Inherit Metab Dis. 2009;32(1):32–9. 18956251 10.1007/s10545-008-0952-4 PMC3633220

[14] Matalon R, Michals-Matalon K, Bhatia G, Grechanina E, Novikov P, McDonald JD, et al. Large neutral amino acids in the treatment of phenylketonuria (PKU). J Inherit Metab Dis. 2006;29(6):732–8. 16988900 10.1007/s10545-006-0395-8

[15] Levy HL, Milanowski A, Chakrapani A, Cleary M, Lee P, Trefz FK, et al. Efficacy of sapropterin dihydrochloride (tetrahydrobiopterin, 6R-BH4) for reduction of phenylalanine concentration in patients with phenylketonuria: a phase III randomised placebo-controlled study. Lancet. 2007;370(9586):504–10. 17693179 10.1016/S0140-6736(07)61234-3

[16] Harding CO, Amato RS, Stuy M, Longo N, Burton BK, Posner J, et al. Pegvaliase for the treatment of phenylketonuria: A pivotal, double-blind randomized discontinuation Phase 3 clinical trial. Mol Genet Metab. 2018;124(1):20–6. 29628378 10.1016/j.ymgme.2018.03.003

[17] Doğum İstatistikleri, 2021 (In Turkish; Birth statistics,2021) [Available from: https://data.tuik.gov.tr/Bulten/Index?p=Dogum-Istatistikleri-2021-45547.

[18] Program İstatistikleri - Yenidoğan Metabolik ve Endokrin Hastalık Taraması (In Turkish; Program Statistics - Newborn Screening for Metabolic and Endocrine Disease) [Available from: https://hsgm.saglik.gov.tr/depo/birimler/cocuk-ergen-sagligi-db/Dokumanlar/Istatistikler/NTP.pdf.

[19] Yenidoğan Metabolik ve Endokrin Hastalık Tarama Programı (In Turkish; Newborn Screening for Metabolic and Endocrine Disease Program) [Available from: https://hsgm.saglik.gov.tr/tr/tarama-programlari/ntp.html.

[20] Pediatrik beslenme ve metabolizma klinikleri (In Turkish; Pediatric nutrition and metabolic clinics) [Available from: https://hsgm.saglik.gov.tr/depo/birimler/cocuk-ergen-sagligi-db/Programlar/Pediatrik_Metabolizma_Klinikleri_Listesi.pdf.

[21] Türkiye’de illere göre nüfus (In Turkish; Population of cities in Turkey) [Available from: https://data.tuik.gov.tr/Bulten/Index?p=Adrese-Dayali-Nufus-Kayit-Sistemi-Sonuclari-2023-49684.

[22] Smith I, Beasley MG, Ades AE. Intelligence and quality of dietary treatment in phenylketonuria. Arch Dis Child. 1990;65(5):472–8. 2357082 10.1136/adc.65.5.472 PMC1792169

[23] Giżewska M, MacDonald A, Bélanger-Quintana A, Burlina A, Cleary M, Coşkun T, et al. Diagnostic and management practices for phenylketonuria in 19 countries of the South and Eastern European Region: survey results. Eur J Pediatr. 2016;175(2):261–72. 26350228 10.1007/s00431-015-2622-5 PMC4724370

[24] Kenneson A, Singh RH. Natural history of children and adults with phenylketonuria in the NBS-PKU Connect registry. Mol Genet Metab. 2021;134(3):243–9. 34654619 10.1016/j.ymgme.2021.10.001

[25] Rezabeigi Davarani EMTF, Daneshi S, Sanjari P, Khanjani N, Hushmandi K, Raei M. Assessing the phenylketonuria (PKU) neonatal screening program and the incidence rates of PKU in Kerman County, Iran: health system research. J Pediatr Neonat Individual Med. 2022;11(2): e110217.

[26] Dababneh S, Alsbou M, Taani N, Sharkas G, Ismael R, Maraqa L, et al. Epidemiology of phenylketonuria disease in Jordan: medical and nutritional challenges. Children (Basel). 2022;9(3):402. 35327772 10.3390/children9030402 PMC8947754

[27] Costa RD, Ferreira MFC, Rocha TA, Galera MF. Evaluation of newborn screening in the state of Mato Grosso from 2005 to 2019. Rev Paul Pediatr. 2023;42: e2022161. 37646746 10.1590/1984-0462/2024/42/2022161 PMC10503421

[28] Savli PEM, Guner AE, Tas I. Evaluation of babies with hyperphenylalaninemia diagnosed in the National Newborn Screening Program in Istanbul in 2019. Int J Med Biochem. 2022;5(1):49–53.

[29] Kadıoğlu Yılmaz B, Bağcı Z. Delays in Newborn screening for phenylketonuria from birth to diagnosis and factors affecting this. Children (Basel). 2024;11(5):571. 38790566 10.3390/children11050571 PMC11120001

[30] Al-Sulaiman A, Kondkar AA, Saeedi MY, Saadallah A, Al-Odaib A, Abu-Amero KK. Assessment of the knowledge and attitudes of saudi mothers towards newborn screening. Biomed Res Int. 2015;2015: 718674. 26543864 10.1155/2015/718674 PMC4620516

[31] Franková V, Dohnalová A, Pešková K, Hermánková R, O’Driscoll R, Ješina P, et al. Factors influencing parental awareness about newborn screening. Int J Neonatal Screen. 2019;5(3):35. 33072994 10.3390/ijns5030035 PMC7510194

[32] Kasem A, Razeq NMA, Abuhammad S, Alkhazali H. Mothers’ knowledge and attitudes about newborn screening in Jordan. J Community Genet. 2022;13(2):215–25. 35013912 10.1007/s12687-021-00572-x PMC8745547

[33] Kadiroğlu T, Altay G, Akay G, Can BÇ. Identification of maternal attitudes and knowledge about newborn screenings: a Turkey sample. J Commun Genet. 2023;14(6):555–64. 10.1007/s12687-023-00659-7 PMC10725403 37535305

[34] Koracin V, Loeber JG, Mlinaric M, Battelino T, Bonham JR, Groselj U. Global impact of COVID-19 on newborn screening programmes. BMJ Glob Health. 2022;7(3):e007780. 10.1136/bmjgh-2021-007780 PMC8895417 35236661

[35] Cakmak S YY, Kanburoğlu MK. COVID-19 Pandemisinin Yenidoğan Tarama Programı Hizmetleri Üzerine Etkileri. (Article in Turkish; The Effects of the COVID-19 Pandemic on Newborn Screening Program Services) SABD. 2022;12(2):224–9.

[36] Esmeray 0 ÖC, Çetin H, Şimşek EE. Bir eğitim aile sağlığı merkezinin COVID-19 pandemi deneyiminin incelenmesi. (Article in Turkish; Evaluation of the COVID-19 pandemia experience of a family health center) Acta Med Nicomedia. Haziran 2021;4(2):56–63.

[37] Selamioğlu A KM, Balcı MC, Burmacı Can N, Gökçay, GF. Covid- 19 Pandemisinin Fenilketonüri Yenidoğan Tarama Programı Üzerine Etkileri Presented at 2nd International Congress of Pediatrics and Gynecology; 21 -24October 2021 Antalya, Türkiye.

[38] 2018 Nüfus ve Sağlık Araştırması (in Turkish: The 2018 Turkey Demographic and Health Survey Hacettepe University Institute of Population Studies): Hacettepe Üniversitesi Nüfus Etütleri Enstitüsü, T.C. Cumhurbaşkanlığı Strateji ve Bütçe Başkanlığı ve Tübitak; [Available from: http://www.sck.gov.tr/wp-content/uploads/2020/08/TNSA2018_ana_Rapor.pdf.

[39] İstatistiklerle Aile, 2023 (in Turkish; Family Statistics, 2023) [Available from: https://data.tuik.gov.tr/Bulten/Index?p=Istatistiklerle-Aile-2023-53784.

[40] Temaj GNN, Sayer JA. The impact of consanguinity on human health and disease with an emphasis on rare diseases. J Rare Dis. 2022. 10.1007/s44162-022-00004-5.

[41] Toktaş İ, Sarıbaş S, Canpolat S, Erdem Ö, Özbek MN. Evaluation of patients diagnosed with phenylketonuria and biotinidase deficiency by the newborn screening program: a ten-year retrospective study. Turk J Pediatr. 2022;64(6):985–92. 36583880 10.24953/turkjped.2022.467

[42] Oz S. Çukurova Üniversitesi Pediatrik Metabolizma ve Beslenme Bilim Dalı’nda izlenen hiperfenilalaninemili hastaların demografik, klinik ve genetik özellikleri (Dissertation in Turkish; Demographic, clinical and genetic characteristics of patients with hyperphenylalaninemia followed-up in Çukurova University Department of Pediatric Metabolism and Nutrition) Adana Turkey; Cukurova University, 2018.

[43] Shirzadeh T, Saeidian AH, Bagherian H, Salehpour S, Setoodeh A, Alaei MR, et al. Molecular genetics of a cohort of 635 cases of phenylketonuria in a consanguineous population. J Inherit Metab Dis. 2018;41(6):1159–67. 30159852 10.1007/s10545-018-0228-6

[44] Ozgüç M, Ozalp I, Coşkun T, Yilmaz E, Erdem H, Ayter S. Mutation analysis in Turkish phenylketonuria patients. J Med Genet. 1993;30(2):129–30. 8445616 10.1136/jmg.30.2.129 PMC1016269

