# Supplementary material for: A comprehensive integrated disease management program for phenylketonuria (IDMP-PKU) from Türkiye: rationale, design and patient characteristics
Source: Orphanet J Rare Dis. 2025 Aug 1;20:394. doi: 10.1186/s13023-025-03702-7 (PMC12317577; doi:10.1186/s13023-025-03702-7)
Supplement: Supplementary file 1 — Additional file 1. [file 13023_2025_3702_MOESM1_ESM.docx]

| **2019** |
| --- |
| **2020** |
| **2021** |
| **2022**  Investigator meeting **January 20^th^** |
| **2023** |
| **2024** |

The first COVID-19 case in Turkey was reported in March 2020, which caused a delay in preparation of study documents, ethics committee approval and patient enrolment.

Authors’ meeting (interim analysis publication) **May 7^th^ - 8^th^**

Data cut-off date for interim analysis **April 30^th^**

Interim analysis **January - March**

Data cleaning **May - December**

Authors’ meeting (interim analysis publication) **March 28^th^**

Authors’ meeting (interim analysis publication) **December 18^th^**

Health authority approval for protocol amendment **June 6^th^**

Approval of protocol amendment by the ethics committee **May 12^th^**

Manuscript writing **March - May**

First patient enrollment by Cukurova University study site **January 5th**

First patient enrollment in the study (by Hacettepe University study site) **May 20^th^**

Investigator meeting **February 17^th^**

Clinical Research Ethics Committee approval (Cukurova University) **November 19^th^**

Preparation of the research protocol and other study-related documents

Creation of the study database **September 2019 to September 2020**

Preparation of final study documents for Ethics Committee submission **October**

Health authority approval **December 14^th^**

Investigator meeting **October 6^th^**

Advisory board meeting: PKU-IDMP (background, unmet needs, rationale, design, setting, data collection, protocol development) March **3-4^th^**

Advisory board meeting: PKU-IDMP - protocol development **March 25^th^**

Meeting with key medical experts for nutritional follow-up and neurocognitive function assessments **July 10^th^**

Follow-up meeting for neurocognitive function assessments **August 8^th^**

First patient enrollment by Istanbul University study site **June 30^th^**

Investigator meeting **December 7^th^**
