# Supplementary material for: A comprehensive integrated disease management program for phenylketonuria (IDMP-PKU) from Türkiye: rationale, design and patient characteristics
Source: Orphanet J Rare Dis. 2025 Aug 1;20:394. doi: 10.1186/s13023-025-03702-7 (PMC12317577; doi:10.1186/s13023-025-03702-7)
Supplement: Supplementary file 2 — Additional file 2. [file 13023_2025_3702_MOESM2_ESM.docx]

**ST 5 - Number of children with PKU and total number of children in the families**

|  | | **Number of children with HPA/PKU in a family** | | | | | | | **Number of families** |
| --- | --- | --- | --- | --- | --- | --- | --- | --- | --- |
|  |  | **1** | **2** | **3** | | **4** | **5** | |  |
| **Total number of children in a family** | **1** | 344 (100) | - | - | | - | - | | 344 |
|  | **2** | 451 (77.6) | 130 (22.4) | - | | - | - | | 581 |
|  | **3** | 222 (69.2) | 85 (26.5) | 14 (4.4) | | - | - | | 321 |
|  | **4** | 69 (60) | 39 (33.9) | 6 (5.2) | | 1 (0.9) | - | | 115 |
|  | **5** | 16 (69.6) | 6 (26.1) | - | | - | 1 (4.3) | | 23 |
|  | **6** | 3 (37.5) | 5 (62.5) | - | | - | - | | 8 |
|  | **7** | 2 (50) | 1 (25) | 1 (25) | | - | - | | 4 |
|  | **8** | 1 (100) | - | - | | - | - | | 1 |
|  | **10** | 1 (100) | - | - | | - | - | | 1 |
| **Number of families** | | 1109 | 266 | 21 | | 1 | 1 | | 1398 |
|  | | | | | | | | | |
| Number of children in a family - Number of children with HPA/PKU in a family | | | | | Correlations (Spearman's rho) | | | | |
|  |  |  |  |  | r | | | p | |
|  |  |  |  |  | 0.315 | | | < 0.001 | |
