# Supplementary material for: A comprehensive integrated disease management program for phenylketonuria (IDMP-PKU) from Türkiye: rationale, design and patient characteristics
Source: Orphanet J Rare Dis. 2025 Aug 1;20:394. doi: 10.1186/s13023-025-03702-7 (PMC12317577; doi:10.1186/s13023-025-03702-7)
Supplement: Supplementary file 3 — Additional file 3. [file 13023_2025_3702_MOESM3_ESM.docx]

**ST 2- Relationship between number of children with HPA/PKU in a family and parental consanguinity**

|  | |  |  | Parental consanguinity | | Number of families | p value |
| --- | --- | --- | --- | --- | --- | --- | --- |
|  | |  |  | Yes | No |  |  |
| Number of children with HPA/PKU in a family | |  | 1 | 439 (40.2) | 654 (59.8) | 1093 | <0.001* |
|  |  |  | 2 | 145 (54.9) | 119 (45.1) | 264 |  |
|  |  |  | 3 | 16 (76.2) | 5 (23.8) | 21 |  |
|  |  |  | 4 | - | 1 (100) | 1 |  |
|  |  |  | 5 | - | 1 (100) | 1 |  |
|  | Total | | | 600 (43.5) | 780 (56.5) | 1380 |  |

**HPA:** Hyperphenylalaninemia; **PKU:** Phenylketonuria

*Kruskal Wallis test
