# Supplementary material for: A comprehensive integrated disease management program for phenylketonuria (IDMP-PKU) from Türkiye: rationale, design and patient characteristics
Source: Orphanet J Rare Dis. 2025 Aug 1;20:394. doi: 10.1186/s13023-025-03702-7 (PMC12317577; doi:10.1186/s13023-025-03702-7)
Supplement: Supplementary file 4 — Additional file 4. [file 13023_2025_3702_MOESM4_ESM.docx]

**ST 3 - Distribution of patients diagnosed with NBS by time to diagnosis (days) (n=1,313)**

| **Time interval** | **n (%)** | **Mean±SD** |
| --- | --- | --- |
| First 7 days of life | 74 (5.6) | 5.5±1.8 |
| 8-15 days | 354 (27) | 11.7±2.2 |
| 16-30 days | 510 (38.8) | 21.8±4.3 |
| 1-3 months | 312 (23.8) | 48.6±15.7 |
| 3-6 months | 37 (2.8) | 119.6±27 |
| 6 months- 1 year | 11 (0.8) | 242.3±53.6 |
| 1-3 years | 7 (0.5) | 686.3±279.8 |
| 3-6 years | 3 (0.2) | 1578.7±228.2 |
| 6-10 years | 3 (0.2) | 2938.3±514.7 |
| 11-18 years | 2 (0.2) | 4761±526.1 |
