# Supplementary material for: A comprehensive integrated disease management program for phenylketonuria (IDMP-PKU) from Türkiye: rationale, design and patient characteristics
Source: Orphanet J Rare Dis. 2025 Aug 1;20:394. doi: 10.1186/s13023-025-03702-7 (PMC12317577; doi:10.1186/s13023-025-03702-7)
Supplement: Supplementary file 5 — Additional file 5. [file 13023_2025_3702_MOESM5_ESM.docx]

**ST 10- Phenotypes and untreated blood Phe levels of patients who underwent NBS by year of diagnosis**

|  | Phenotypes classified based on untreated Phe levels (µMol/L)  Median (IQR 25-75) | | | |  | |
| --- | --- | --- | --- | --- | --- | --- |
| Year | **HPA** (120 ≤ x <360) | **Mild PKU**  (360≤ x < 600) | **Moderate PKU**  (600≤ x <1200) | **Classical PKU**  (1200≤ x) | | p value |
| 1985-2006  (Before national NBS) | 25.5 (15.5-61.3) | 31.5 (10.8-71) | 31.5 (18.3-65.8) | 32 (17-59) | 0.987^a^ | |
| As of 2007  (National NBS) | 23 (16-32) | 17 (12-25) | )  17 (12-28) | 16 (12-22) | <0.001^a^ | |

**HPA:** Hyperphenylalaninemia; **IQR:** Interquartile range; **NBS:** Newborn screening; **PKU:** Phenylketonuria; **SD:** Standard deviation

^a^ Kruskal Wallis test
