# Supplementary material for: A comprehensive integrated disease management program for phenylketonuria (IDMP-PKU) from Türkiye: rationale, design and patient characteristics
Source: Orphanet J Rare Dis. 2025 Aug 1;20:394. doi: 10.1186/s13023-025-03702-7 (PMC12317577; doi:10.1186/s13023-025-03702-7)
Supplement: Supplementary file 6 — Additional file 6. [file 13023_2025_3702_MOESM6_ESM.docx]

**ST 5 - Relationship between time to diagnosis and maternal educational level**

|  | | **n(%)** | **(Median) (Q25-Q75)** | **p* value** |
| --- | --- | --- | --- | --- |
| **Maternal education**  (n=1359) | No education | 80 (5.9) | 25.5 (15.3-56.5) | **<0.001** |
|  | Primary/middle school | 640 (47.1) | 25 (14-55) |  |
|  | High school | 364 (26.8) | 19 (13-31) |  |
|  | University | 247 (18.2) | 20 (13-30) |  |
|  | Master | 28 (2.1) | 19 (11-28.8) |  |

*Kruskal Wallis test. Bonferroni correction and pairwise comparisons with the Mann-Whitney U test were made to detect the group that made the difference (No Education and Primary/middle school education).
