# Supplementary material for: A comprehensive integrated disease management program for phenylketonuria (IDMP-PKU) from Türkiye: rationale, design and patient characteristics
Source: Orphanet J Rare Dis. 2025 Aug 1;20:394. doi: 10.1186/s13023-025-03702-7 (PMC12317577; doi:10.1186/s13023-025-03702-7)
Supplement: Supplementary file 7 — Additional file 7. [file 13023_2025_3702_MOESM7_ESM.docx]

**ST 6_ Association between PAH variants and degree of parental consanguinity**

| Degree of parental consanguinity | | Allelic variant | | Total | p value |
| --- | --- | --- | --- | --- | --- |
|  |  | Homozygous | Compound heterozygous |  |  |
| First cousin | | 194 (84) | 37 (16) | 231 | <0.001 |
| Second cousin | | 32 (69.6) | 14 (30.4) | 46 |  |
| Distant relative | | 62 (63.3) | 36 (36.7) | 98 |  |
| No consanguinity | Same village | 24 (31.2) | 53 (68.8) | 77 |  |
|  | Different village | 67 (14.7) | 388 (85.3) | 455 |  |

*Chi square test
