# Supplementary material for: A comprehensive integrated disease management program for phenylketonuria (IDMP-PKU) from Türkiye: rationale, design and patient characteristics
Source: Orphanet J Rare Dis. 2025 Aug 1;20:394. doi: 10.1186/s13023-025-03702-7 (PMC12317577; doi:10.1186/s13023-025-03702-7)
Supplement: Supplementary file 8 — Additional file 8. [file 13023_2025_3702_MOESM8_ESM.docx]

**ST-7 Common *PAH* mutations (allelic frequency >5%) in the study population.**

| **Description of mutation** | | **Allelic frequency**  **n (%)** |
| --- | --- | --- |
| **Protein variant** | **cDNA aberration** |  |
| IVS-10-11G>A | c.1066-11G>A | 450 /1976 (22.8) |
| p.Arg261Gln | c.782G>A | 181/1976 (9.2) |
| p.Ala300Ser | c.898G>T | 123/1976 (6.2) |
| p.Leu48Ser | c.143T>C | 111/1976 (5.6) |
| p.Pro281Leu | c.842C>T | 104/1976 (5.3) |
